# Prevalence and Antimicrobial Resistance of ESBL *E. coli* in Early Broiler Production Stage and Farm Environment in Lithuania

**DOI:** 10.3390/microorganisms13020425

**Published:** 2025-02-15

**Authors:** Beatrice Kasparaviciene, Aleksandr Novoslavskij, Jurgita Aksomaitiene, Jurate Stankeviciene, Neringa Kasetiene, Romualdas Sinkevicius, Mindaugas Malakauskas

**Affiliations:** 1 Department of Food Safety and Quality, Faculty of Veterinary Medicine, Lithuanian University of Health Sciences, Tilzes Str. 18, LT-47181 Kaunas, Lithuania; beatrice.kasparaviciene@lsmu.lt (B.K.); jurgita.aksomaitiene@lsmu.lt (J.A.); jurate.stankeviciene1@lsmu.lt (J.S.); neringa.kasetiene@lsmu.lt (N.K.); mindaugas.malakauskas@lsmu.lt (M.M.); 2 Department of Animal Nutrition, Faculty of Animal Sciences, Lithuanian University of Health Sciences, Tilzes Str. 18, LT-47181 Kaunas, Lithuania; romualdas.sinkevicius@lsmu.lt

**Keywords:** ESBL *E. coli*, prevalence, antimicrobial resistance, broilers, farm environment

## Abstract

*Escherichia coli*, a major opportunistic pathogen in chickens, poses a serious threat to poultry production and public health via potential zoonotic transmission of ESBL-producing strains. Therefore, this study aimed to emphasize broilers as early carriers of ESBL *E. coli* and provide deeper insights into antimicrobial resistance of these bacteria. Prevalence and antimicrobial resistance (MIC) testing of ESBL *E. coli* in cloacal and environmental samples from one-day-old and five-day-old broilers was conducted on three different growth cycles from a conventional poultry farm in Lithuania. Confirmed prevalence of ESBL *E. coli* in cloacal samples ranged from 0% to 57.5%, and in environmental swabs from 0% to 25%. All 102 ESBL *E. coli* isolates were susceptible to meropenem, imipenem, fosfomycin, and colistin. However, 93.14% of the strains were resistant to ceftriaxone (89.06–100%, depending on bacteria isolation source), 97.06% to ciprofloxacin (95.31–100%), and 66.67% to tetracycline (26.09–100%). Additionally, 80.39% of ESBL *E. coli* strains exhibited multidrug resistance. In total, 23 different antimicrobial resistance profiles were confirmed, with CRO/AMS/AUG/CIP/SXT/TE and CRO/CIP being the most common, detected in 18 of the 102 strains. The detection of widespread antimicrobial-resistant ESBL *E. coli* in five-day-old broilers emphasizes the need to implement control strategies early in the broiler production cycle.

## 1. Introduction

Extended-spectrum β-lactamase (ESBL) producing *Escherichia coli* is commonly reported in humans and food-producing animals, including chickens [1,2]. According to the EFSA and ECDC report, the prevalence of presumptive ESBL-producing *E. coli* in broilers across European countries ranged between 0.7% and 70.5% in 2021–2022 [3]. Similar findings have been reported in studies from China and Bangladesh, where the prevalence of ESBL *E. coli* in broilers of different ages varies from 5% to 76.8% [4,5]. In Lithuania, the prevalence of ESBL-producing *E. coli* in broilers was confirmed at 39.3% in 2022 [3]. Both horizontal and vertical transmission of ESBL *E. coli* can occur in broilers, with bacteria detected as early as one day of age, and the number of infected birds increasing as the broiler grows [6,7].

Extended-spectrum β-lactamase producing *E. coli* produce enzymes that inactivate β-lactams, as well as first-, second-, and third-generation cephalosporins, and aztreonam, ref. [8] which are extensively used to manage infections in both humans and animals [9]. Antibiotic exposure and selection pressure leads to antimicrobial resistance, and the widespread usage of cephalosporins is linked to the spread of bacteria that produce ESBLs [10]. Furthermore, if such irrational antibiotic use continues, infections with Gram-negative bacteria are expected to increase the antibiotic resistance rates in the near future, resulting in higher morbidity and mortality [11]. Noteworthy, the majority of ESBL *E. coli* are co- or multidrug-resistant (MDR) displaying resistance to three or more antibiotic classes including such as fluoroquinolones, aminoglycosides, and trimethoprim-sulfamethoxazole [1,11].

It is important to evaluate the role of food animals as reservoirs and spreaders of ESBL producers via the food production chain. Noteworthy, *E. coli* is the leading opportunistic pathogen in chickens and has the potential for zoonotic transmission to humans through the food chain. Therefore, ESBL-producing *E. coli* strains present a substantial threat to both poultry production and public health [1,12,13,14].

The aim of this study was to investigate young broilers as a carriers of ESBL *E. coli* at the early broiler production stages and to expand the existing knowledge of phenotypic antimicrobial resistance patterns of this bacterium.

## 2. Materials and Methods

### 2.1. Ethics Statement

This study experiments were approved by Lithuanian University of Health Sciences (LSMU) Bioethics Centre (No 2024-BEC3-T-012).

### 2.2. Broilers and Husbandry

The investigation into early-stage ESBL *E. coli* prevalence and antimicrobial resistance was conducted in a conventional poultry production farm in Lithuania. Approximately 20,000 Ross 308 broilers (stocking density 19–20 birds/m^2^) were reared in each poultry house. The birds were floor-reared on peat-moss bedding under temperatures ranging from 31 °C to 33 °C. Broilers were provided with free access to antibiotic-free feed and drinking water during their raising period. All broilers were raised under identical conditions in each production cycle. The all-in-all-out management system was implemented in the tested poultry farm, with deep cleaning and disinfection procedures performed before the beginning of a new broiler production cycle .

### 2.3. Sample Collection

In total 606 samples were collected from poultry farm at three broiler growth cycles including cloacal swabs from broilers (*n* = 480), swabs from flock environment (*n* = 72; swab samples from feeders, drinking line cups, and worker boots), drinking system water samples (*n* = 24), and peat-moss bedding samples (*n* = 30) collected before the chickens were introduced to the farm environment. All samples were collected on day 0 (one-day-old chickens are introduced to the farm) and day 5 (five-day-old broilers). Cycle I represents the broiler growth period between April and May 2023; Cycle II represents the period between October and November 2023; and Cycle III represents the period between February and March, 2024. During each broiler growth cycle, two broiler flocks were tested. In total, 80 cloacal swabs from broilers, 12 swabs from flock environment, and 4 water samples were collected on day 0 and day 5, respectively.

Each cloacal swab was placed in glass tubes with 5 mL of buffered peptone water (BPW) (Liofilchem, Roseto degli Abruzzi, Italy). Farm environment swabs were collected using a transport medium Transwab^®^ (Medical Wire and Equipment Co., Corsham, UK). Water samples were aseptically collected from the end of the broiler drinking line using sterile 250 mL glass bottles. Bedding samples were aseptically collected into sterile plastic bags (approx. 100 g).

All collected samples were labeled with identification numbers and the date of collection and delivered to the laboratory within 4 h at 4–6 °C.

### 2.4. Isolation of Presumptive ESBL E. coli

Upon arrival at the laboratory environmental, swabs and 10 g of bedding samples were diluted in 5 mL and 90 mL of BPW (Liofilchem, Italy), respectively. All prepared samples, including cloacal swab samples, were homogenized and incubated at 37 °C for 24 h. For the detection of cefotaxime-resistant *Escherichia coli*, 10 µL of each enriched sample was spread onto Tryptone Bile X-glucuronide (TBX) agar (Biolife, Milan, Italy) supplemented with 1 mg/L cefotaxime (TBX + CTX). Water samples were analyzed for *E. coli* using the membrane filtration method in accordance with LST EN ISO 9308-1:2014 [15] guidelines. Bacterial colonies appearing dark blue or violet color on Chromogenic Coliform Agar (Biolife, Italy), were transferred to TBX + CTX agar and incubated at 37 °C for 18–24 h. A sample was considered positive for cefotaxime-resistant *E. coli* if at least one green colony was observed on TBX + CTX agar.

Presumptive ESBL *E. coli* isolates were further purified using the streak plate method on TBX agar and subsequently cultured overnight on Tryptic Soy Agar (TSA) (Liofilchem, Italy) at 37 °C. These isolates were then stored in Brain Heart Infusion broth (Liofilchem, Italy) supplemented with glycerol at −80 °C for further analysis.

### 2.5. Molecular Confirmation of the Presumptive ESBL E. coli

In total, 1 μL loopful of bacteria grown at 37 °C on TSA plates for 24 h were suspended in Eppendorf tubes containing 200 μL of PrepMan Ultra Sample Preparation Reagent (PrepMan™ Ultra, Applied Biosystems, Waltham, MA, USA). Template DNA extraction for PCR was carried out following the instructions of the supplier, including heating of bacterial suspension at 99 °C for 10 min, and centrifugation at 14,000 rpm for 5 min. The confirmation of *E. coli* was carried out by multiplex PCR (mPCR) using the flanking region of *uspA* (the universal stress protein) and *uidA* (β-glucuronidase) gene amplification as described by Godambe et al. [16].

### 2.6. Screening and Confirmation of ESBL Production

Extended-spectrum β-lactamase-producing *Escherichia coli* confirmation was performed using the disk diffusion test with ceftazidime and cefotaxime+/− clavulanic acid disks based on the guidelines of The European Committee on Antimicrobial Susceptibility Testing (EUCAST, 2017) [17]. *Escherichia coli* NCTC 13351 and 25922 *E. coli* ATCC^®^ 25922 were used as a positive and negative control, respectively.

### 2.7. ESBL E. coli Phenotypic Antibiotic Susceptibility Testing

The antibiotic susceptibility of ESBL-producing *E. coli* isolates was assessed by determining the minimum inhibitory concentration (MIC) using the gradient strip diffusion method with ETEST antibiotic strips (Liofilchem, Italy). The test was performed using 13 antibiotics, including ceftriaxone (0.016–256 μg/mL) (CRO), ampicillin/sulbactam (0.016–256 μg/mL) (AMS), amoxicillin/clavulanate (0.016–256 μg/mL) (AUG), ciprofloxacin (0.002–32 μg/mL) (CIP), meropenem (0.002–32 μg/mL) (MRP), imipenem (0.002–32 μg/mL) (IMI), gentamicin (0.064–1024 μg/mL) (GEN), amikacin (0.016–256 μg/mL) (AK), trimethoprim/sulfamethoxazole (0.002–32 μg/mL) (SXT), tetracycline (0.016–256 μg/mL) (TET), aztreonam (0.016–256 μg/mL) (ATM), fosfomycin (0.016–256 μg/mL) (FOS), and colistin (0.016–256 μg/mL) (CS). Bacterial suspension with a turbidity equivalent to 0.5 McFarland standard was evenly dispensed on the surface of Mueller–Hinton (MH) agar (OXOID, Basingstoke, UK) plates using a sterile cotton swab. The phenotypic characterization of the analyzed strains was performed based on minimum inhibitory concentration (MIC) breakpoints in accordance with the European Committee on Antimicrobial Susceptibility Testing (EUCAST) guidelines (2024) [18], with the exception of tetracycline, for which MIC values were interpreted according to the 2024 Clinical and Laboratory Standards Institute (CLSI) guidelines [19]. Isolates exhibiting resistance to three or more classes of antimicrobial agents were classified as multidrug-resistant (MDR). *Escherichia coli* ATCC^®^ 25922 was used as a control strain.

### 2.8. Statistical Analysis

The study’s findings were evaluated using Microsoft Office Excel 2016 (Microsoft Corp., Redmond, WA, USA) and the IBM SPSS Statistics 29.0.1 software package (IBM Corp, Armonk, NY, USA). The Chi-Square (χ^2^) test and Fisher’s exact test were used to compare ESBL *E. coli* prevalence rates and AMR differences between different broiler production cycles and ESBL *E. coli* isolate sources in different combinations. Differences were considered significant if *p* < 0.05.

## 3. Results

### 3.1. Prevalence of ESBL E. coli

Extended-spectrum β-lactamase-producing *E. coli* was not detected in broiler cloacal and farm environment swab samples on day 0 across cycles I–III. Moreover, bacteria were absent from bedding samples on day 0. However, ESBL *E. coli*-positive broiler cloacal and farm environment swab samples were found on day 5. In total ESBL *E. coli* was isolated from 75 (27.2%) out of the 276 samples collected on day 5. Overall, ESBL *E. coli* was detected in 29.17% of broiler cloacal swab samples and 13.88% of environmental swab samples collected from the farm. However, ESBL *E. coli* was not detected in the drinking water system samples collected on day 0 and day 5.

**Figure 1:**
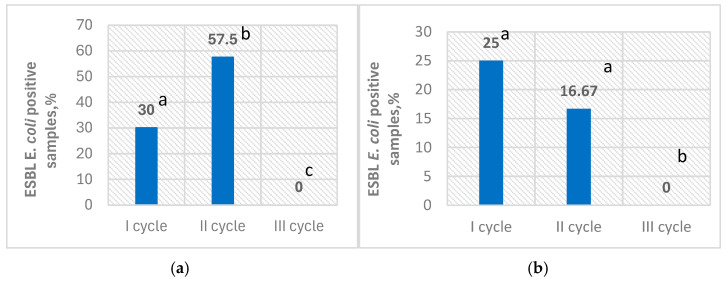
Prevalence of ESBL *E. coli* during different production cycles on day 5; ESBL *E. coli*-positive broiler cloacal swab samples (%) (**a**); ESBL *E. coli*-positive farm environment swab samples (%) (**b**); values on the columns noted by different superscript letters are significantly different (*p* < 0.05).

Prevalence of ESBL *E. coli* in broiler cloacal swab samples and farm environment swab samples ranged from 0% up to 57.5% and 0% up to 25%, across the tested broiler growth cycles, respectively (Figure 1).

### 3.2. Antimicrobial Susceptibility

A total of 102 *E. coli* isolates from broiler cloacal swabs and the farm environment collected during broiler growth cycles I–II were tested for antimicrobial susceptibility to 13 different antibiotics. All ESBL *E. coli* isolates (15 environmental isolates and 87 cloacal isolates) were susceptible to imipenem (IMI), fosfomycin (FOS), colistin (CS), and meropenem (MRP). In total, 93,14% of tested ESBL *E. coli* isolates were resistant to ceftriaxone (CRO) (89.06–100%, depending on bacteria isolation source); 58.82% resistant to ampicillin/sulbactam (AMS) (56.52–66.67%); 40.20% resistant to amoxicillin/clavulanate (AUG) (0–66.67%); 97.06% resistant to ciprofloxacin (CIP) (95.31–100%); 5.88 resistant to gentamicin (CN) (0–33.33%); 4.90% resistant to amikacin (AK) (0–22.22%); 34.31% resistant to trimethoprim/sulfamethoxazole (SXT) (22.22–50%); 66.67% resistant to tetracycline (TE) (26.09–100%); and 7.84% resistant to aztreonam (ATM) (0–33.33%) (Table 1).

Extended-spectrum β-lactamase-producing *E. coli* isolated from broiler production cycle II were more often resistant to amoxicillin/clavulanic acid (AUG) and tetracycline (TE) in comparison to bacteria isolated from cycle I (54.29% compared to 9.38%, (*p* < 0.05) and (82.86% compared to 31.25%, (*p* < 0.05)), respectively. Moreover, resistance to gentamicin (CN), amikacin (AK), and aztreonam (ATM) of bacteria isolated from cycle II broiler production was significantly lower (*p* < 0.05) compared to bacteria isolated from cycle I.

In total, 83 (81.37%) ESBL-producing *E. coli* strains exhibited multidrug resistance (strains exhibited resistance to three or more classes of antimicrobial agents) (MDR). During cycle I, 77.78% (7 out of 9) of the ESBL-producing *E. coli* strains isolated from the flock environment demonstrated MDR, while 69.57% (16 out of 23) of the strains from cloacal samples were MDR. In cycle II, all environmental isolates (6 out of 6) exhibited MDR, and 84.37% (54 out of 64) of the ESBL-producing *E. coli* strains from cloacal samples demonstrated MDR. However, statistical analysis revealed no significant differences between the occurrence of multidrug resistance in ESBL *E. coli* isolates of different origin (different production cycles and isolation sources) (*p* > 0.05). Also, worth mentioning is that none of the tested strains exhibited extensive drug resistance (no isolate was resistant to at least one agent in all but two or fewer antimicrobial categories) and none of the collected ESBL *E. coli* strains were pandrug-resistant (resistant to antimicrobials in all antimicrobial categories).

Confirmed minimum inhibitory concentration (MIC) of ESBL-producing *E. coli* bacteria recovered from broiler cloaca (C) and flock environment (E) across two production cycles, depending on antimicrobial tested, varied from 0.004 μg/mL to ≥256 μg/mL (Table 2 and Table 3).

Detected MIC of ceftriaxone (CRO) varied from 2 μg/mL to ≥256 μg/mL (mode—3 μg/mL); ampicillin/sulbactam (AMS) 1.5—≥256 μg/mL (mode—8 μg/mL); amoxicillin/clavulanic acid (AUG) 0.75—≥256 μg/mL (mode—2 μg/mL); ciprofloxacin (CIP) 0.019—≥32 μg/mL (mode—≥32 μg/mL); meropenem (MRP) 0.004—0.016 μg/L (mode—0.008 μg/L); gentamicin (CN) 0.125—128 μg/mL (mode—0.75 μg/mL); amikacin (AK) 1—≥256 μg/mL (mode—3 μg/mL); trimethoprim/sulfamethoxazole (SXT) 0.008—≥32 μg/mL (mode—≥32 μg/mL); tetracycline (TE) 0.094—≥256 μg/mL (mode—24 μg/mL; 64 μg/mL); aztreonam (ATM) 0.5—≥256 μg/mL (mode—1.5 μg/mL); imipenem (IMI) 0.047—0.75 μg/mL (mode—0.094 μg/mL); fosfomycin (FOS) 0.064—0.5 μg/mL (mode—0.19 μg/mL); and colistin (CS) 0.38—1 μg/mL (mode—0.75 μg/mL) (Table 2 and Table 3).

Extended-spectrum β-lactamase-producing *E. coli* strains from cycle II were confirmed to have a higher, most frequently occurring MIC value (mode) of 64 μg/mL for tetracycline compared to the bacteria isolated from cycle I broiler growth (MIC mode of tetracycline—0.75 μg/mL) (Table 3). In the case of amoxicillin/clavulanic acid (AUG), confirmed MICs from cycles I and II were similar (1 μg/mL—≥256 μg/mL; mode—3 μg/mL and 0.75 μg/mL—≥256 μg/mL; mode—2 μg/mL, respectively) (Table 3) regardless of cycle II strains statistically significantly higher (*p* < 0.05) resistance frequency to this antibiotic (Table 1).

Noteworthy, MIC values for amikacin remained consistent across both cycles (1 μg/mL to ≥256 μg/mL, mode 4 μg/mL in cycle I, and 1.5 μg/mL to ≥256 μg/mL, mode 3 μg/mL in cycle II) despite a significant difference in the number of resistant strains detected between the tested broiler growth cycles. Confirmed MIC of gentamicin in bacteria isolated from cycle I ranged from 0.125 μg/mL to 128 μg/mL (mode 0.75 μg/mL), while in cycle II it ranged from 0.25 μg/mL to 2 μg/mL (mode 0.5 μg/mL). MIC of aztreonam in cycle I varied from 0.5 μg/mL to ≥256 μg/mL (mode 1.5 μg/mL), but in cycle II confirmed MIC ranged from 0.5 μg/mL to 38 μg/mL (mode 1.5 μg/mL), indicating a wider MIC range in cycle I although having the same mode (Table 3).

Ciprofloxacin and ceftriaxone resistance rates did not differ significantly between bacterial isolates from different broiler growth cycles (Table 1). However, the MIC distributions showed notable differences (Table 3). In cycle I, the MIC mode for ceftriaxone was four times higher than detected in cycle II (12 μg/mL vs. 3 μg/mL). In contrast, the MIC mode for ciprofloxacin in bacteria isolated from cycle II (≥32 μg/mL) was more than 10 times higher in comparison to detected MIC mode of bacteria isolated from cycle I (3 μg/mL).

### 3.3. Antimicrobial Resistance Profiles Analysis

In total, 23 different antimicrobial resistance profiles were confirmed among 102 ESBL *E. coli* strains (Table 4). The dominant antimicrobial resistance profiles were CRO/AMS/AUG/CIP/SXT/TE and CRO/CIP, each detected in 18 out of 102 *E. coli* isolates (17.65%). Noteworthy, one ESBL *E. coli* strain isolated from broiler cloaca showed resistance to eight different antibiotics with a confirmed CRO/AMS/CIP/CN/AK/SXT/TE/ATM resistance profile. Altogether, 21 AMR profiles were identified among ESBL *E. coli* isolates from broiler cloacal swabs, with the most common CRO/CIP (18.39%), followed by CRO/AMS/AUG/CIP/SXT/TE (17.24%), and CRO/CIP/TE (16.09%). Meanwhile, ESBL *E. coli* isolated from the farm environment shared nine different AMR profiles. Two additional AMR profiles CRO/AMS/CIP/CN/TE/ATM and CRO/CIP/AK were exclusively identified in environmental isolates.

## 4. Discussion

The presence of ESBL-producing *E. coli* in broilers is considered a potential threat to human health, as the substantial evidence confirms its transmission to humans [7,20], and may lead to difficulties in the treatment of human diseases [21]. Several studies conducted in European countries have indicated a high prevalence of ESBL *E. coli* in broilers [22,23] reaching up to 54% in poultry flocks in Sweden [24] and even 69.2% in fattening broilers from Italy [25]. Furthermore, these bacteria can also be found in chickens at elevated levels across different stages of the broiler production pyramid, including (grand) parent stocks [7]. The study’s findings demonstrate the prevalence of ESBL-producing *E. coli* as well as antibiotic resistance patterns in Lithuania’s broiler production system. Extended-spectrum β-lactamase (ESBL)-producing *Escherichia coli* was detected in broiler cloacal and flock environment samples as early as day 5, despite its absence on day 0, indicating that these bacteria can colonize and spread rapidly in the poultry environment. Scientific literature states that one-day-old broilers can inherit ESBL *E. coli* vertically from parental flocks via contaminated eggs. After hatching, the broiler’s gut can become colonized with these bacteria within hours to days, causing variability in the early swab results, with a rapid rise in ESBL *E. coli*-positive samples during the first week of broiler life [7]. The overall prevalence of ESBL *E. coli* in cloacal swabs (29.17%) and farm environment swabs (13.88%) in five-day-old broiler flocks suggests significant environmental contamination and the capacity of these bacteria to persist and spread within broiler farms at early production points. Depending on the broiler growth cycle, we found that the prevalence of ESBL *E. coli* in five-day-old broiler cloacal samples reached up to 57.5%, whereas in the flock environment it reached up to 25%. These findings align with previous studies that have shown that ESBL-producing *E. coli* can be identified in broiler chicks as young as one-day-old or shortly after broilers are introduced to the poultry farm [7,20,26]. Moreover, some scientific literature states that ESBL-producing *E. coli* in one-day-old chicks consistently leads to their presence in the flock, whereas flocks starting ESBL *E. coli* negative may later test positive, and the number of positive samples can fluctuate at the later fattening points [27]. The investigation in a Dutch commercial broiler farm indicated that ESBL *E. coli* were detected on day 2 with a prevalence of 11% in caecal samples, increasing to 72% by day 5 [28]. Improper cleaning and disinfection procedures of the broiler barns can be one of the causes of the high incidence of ESBL-producing *E. coli* [20]. In our study, ESBL *E. coli* were not found in samples collected from the farm environment prior to the introduction of the broilers. In addition, all tested samples of water from the drinking system were negative for ESBL *E. coli*. This alights with a study in Slovenia where findings were the same [27].

Antimicrobial resistance analysis of ESBL *E. coli* strains revealed high resistance to ciprofloxacin and ceftriaxone (97.06% and 93.14%, respectively). Such an extremely high level of resistance to these antimicrobials is concerning due to their use in human medicine. Moreover, these antibiotics based on the Antimicrobial Advice Ad Hoc Expert Group (AMEG) adopted by both EMA’s veterinary medicines committee and human medicines committee, are in category B (“Restrict”). For these antibiotics, the risk to public health resulting from veterinary use needs to be mitigated by specific restrictions [29]. Findings on ESBL *E. coli* resistance to ciprofloxacin and ceftriaxone vary in different studies. For example, a study in Greece reported low (4.4%) ESBL *E. coli* resistance to ceftriaxone [30], while another study revealed that the resistance to ciprofloxacin of ESBL *E. coli* strains from a conventional broiler farm reached 35.19% [31]. Noteworthy is that sensitivity to carbapenems (imipenem, meropenem), fosfomycin, and colistin demonstrates that these antibiotics are still effective against ESBL *E. coli*.

Significant differences in the ESBL *E. coli* resistance frequencies were observed among bacteria isolated from different broiler production cycles. For instance, isolates from cycle II exhibited significantly higher resistance to amoxicillin/clavulanic acid (54.29% vs. 9.38%, *p* < 0.05) and tetracycline (82.86% vs. 31.25%, *p* < 0.05) compared to bacteria isolated from cycle I, suggesting potential temporal variations in antimicrobial selection pressure or management practices. Conversely, lower resistance rates to gentamicin, amikacin, and aztreonam were noted in ESBL *E. coli* isolated from cycle II. Detected MIC analysis of tested ESBL *E. coli* strains revealed differences among confirmed MIC values (mode) for the majority of tested antibiotics and especially tetracycline. These distinctions underscore the dynamic nature of antimicrobial resistance and the impact of farm-specific factors like antimicrobial use and biosecurity measures in parental flocks [32].

Studies on the antimicrobial resistance of ESBL *E. coli* isolated from young broiler cloaca or the environment are currently limited. In total, 82 (80.39%) ESBL-producing *E. coli* strains tested in our study exhibited multidrug resistance (MDR). The number of MDR ESBL *E. coli* varies among the studies, for example, results from a study in Senegal showed that 91.4% of the isolates displayed MDR, but samples were taken from unspecified age broilers ceacal content [33]. Another study in India showed that 38.23% of ESBL *E. coli* from broiler caecum were confirmed as MDR [34]. The wide range of antimicrobial resistance profiles (23 different profiles) seen in ESBL *E. coli* isolates illustrates the heterogeneity of antimicrobial resistance mechanisms in broiler production systems. The dominant profiles include CRO/AMS/AUG/CIP/SXT/TE and CRO/CIP. Noteworthy, two additional AMR profiles, CRO/AMS/CIP/CN/TE/ATM and CRO/CIP/AK, were confirmed for bacteria isolated from the farm environment, indicating circulation of different ESBL *E. coli* in the farm environment. Important is the discovery of a cloacal isolate resistant to eight different antibiotics (CRO/AMS/CIP/CN/AK/SXT/TE/ATM), which raises worries about the spread of widely drug-resistant bacteria through the food chain, posing a threat to public health. These data reveal the complexity of antimicrobial resistance among ESBL *E. coli* strains, highlighting the prevalence of specific resistance profiles. This could be associated with the high use of these antimicrobials in the poultry sector for disease prevention and the treatment of diseases [35].

Internationally, the issue of ESBL *E. coli* in broiler farming is tackled by a combination of regulatory measures, surveillance programs, and research efforts. The European Union has imposed strict measures to prevent AMR in food-producing animals. Notably, the European Union outlawed the use of antibiotics as growth promoters in animal feed on the 1 January 2006, per Regulation (EC) No 1831/2003. This prohibition is intended to limit the selection pressure that contributes to the emergence of resistant bacteria, including ESBL *E. coli* [36,37]. The European Medicines agency’s European Surveillance of Veterinary Antimicrobial Consumption (ESVAC) project monitors veterinary antimicrobials sales in 31 European Union countries, supplying the data to form a new AMR policy. The latest report, released in November 2023, emphasizes a notable reduction in antimicrobials usage in food-producing animals [38]. Also, the European Food Safety Authority (EFSA) monitors AMR trends, including the prevalence and occurrence of ESBL-producing bacteria in the animals intended for food production [3]. Furthermore, international research partnerships aim to comprehend the prevalence, mechanisms, and transmission routes of ESBL *E. coli* in poultry farms [9,39,40].

Broiler producers take various intervention measures to minimize the effects of pathogenic bacteria, including ESBL *E. coli*, at the farm level. For example, management of the environmental stressors in the broiler flocks [41] and biosecurity measures, such as proper cleaning and disinfection procedures of the broiler farm facilities [42], transport vehicles, worker hands, boots, or other work equipment can help to prevent the introduction and spread of these bacteria [39]. Furthermore, vaccination against *E. coli* can minimize the occurrence and severity of these bacteria-caused infections, however it has some limitations [43]. Another potential approach for decreasing the impact of pathogenic bacteria on broilers is the use of competitive exclusion products which can outcompete pathogens, thereby reducing their abundance in the gut [23].

## 5. Conclusions

Our study results revealed the alarmingly high prevalence of resistant ESBL-producing *E. coli* in apparently healthy five-day-old chickens and the farm environment. The high resistance rates to key antibiotics, such as ciprofloxacin, ceftriaxone, and tetracycline, along with the widespread occurrence of multidrug-resistant strains, underscore a potential risk to poultry production and human health. These results emphasize the need for the strategies to help control ESBL *E. coli* spread in the early stages of broiler production and minimize its impact on both poultry and human health.

## Figures and Tables

**Table 1:** Antimicrobial resistance results of ESBL *E. coli* strains isolated from different broiler production cycles and different isolation sources.

Antibiotic	Percentage of ESBL. *E. coli* Resistant to Tested Antimicrobials								
	Cycle 1			Cycle 2			In total		
	E	C	In Total	E	C	In Total	E	C	In Total
**CRO**	100	100	100	100	89.06	90.00	100	91.95	93.14
**AMS**	66.66	56.52	59.38	66.67	57.81	58.57	66.67	57.47	58.82
**AUG**	0.00	13.04	9.38	66.67	54.69	54.29 *	26.67	42.53	40.20
**CIP**	100	100	100	100	95.31	95.71	100	96.55	97.06
**MRP**	0	0	0	0	0	0	0	0	0
**CN**	33.33	13.04	18.75	0	0	0 *	20	3.45	5.88
**AK**	22.22	8.70	12.50	0	1.56	1.43 *	13.33	3.45	4.90
**SXT**	22.22	26.09	25.00	50	37.50	38.57	33.33	34.48	34.31
**TE**	44.44	26.09	31.25	100	81.25	82.86 *	66.67	66.67	66.67
**ATM**	33.33	17.39	21.88	0	3.13	1.43 *	20.00	5.75	7.84
**IMI**	0	0	0	0	0	0	0	0	0
**FOS**	0	0	0	0	0	0	0	0	0
**CS**	0	0	0	0	0	0	0	0	0

C—bacteria isolated from cloacal swab samples. E—bacteria isolated from environmental swab samples. CRO—Ceftriaxone. AMS—Ampicillin/sulbactam. AUG—Amoxicillin/clavulanic acid. CIP—Ciprofloxacin. MRP—Meropenem. CN—Gentamicin. AK—Amikacin. SXT—Trimethoprim/sulfamethoxazole. TE—Tetracycline. ATM—Aztreonam. IMI—Imipenem. FOS—Fosfomycin. CS—Colistin. *—difference between resistance against antimicrobials on different cycles is significant, *p* < 0.05.

**Table 2:** MIC data on ESBL *E. coli* resistance to meropenem (MRP), trimethoprim/sulfamethoxazole (SXT), imipenem (IMI), fosfomycin (FOS), and colistin (CS). C—number of ESBL *E. coli* isolated from cloacal swab samples. E—number of ESBL *E. coli* isolated from environmental swab samples. (I)—ESBL *E. coli* isolated from broiler production cycle I. (II)—ESBL *E. coli* isolated from broiler production cycle II. No color background—sensitive. Gray color background—resistant.

Antibiotic (μg/mL)	MRP	SXT	IMI	FOS	CS
0.004	2C (I); 1E (I); 2C (II);				
0.006	6C (I); 2E (I); 21C (II); 2E (II);				
0.008	10C (I); 4E (I); 32C (II); 3E (II);	3C (II);			
0.012	4C (I); 2E (I); 8C (II); 1E (II);	5C (II);			
0.023		1C (II); 1E (II);			
0.047		2E (I);	1C (II);		
0.094		5C (I); 2E (I); 11C (II); 2E (II);	11C (I); 9E (I); 26C (II); 4E (II);	5C (II);	
0.125		5C (I); 4C (II);	5C (I); 2E (I); 21C (II); 2E (II);	10C (I); 2E (I); 7C (II);	
0.19		2C (I);	1C (I); 5C (II);	9C (I); 2E (I); 34C (II); 1E (II);	
0.25			1C (II);	4C (I); 4E (I); 13C (II); 4E (II);	
0.38				1E (I); 4C (II);	1C (II);
0.5				1E (II);	9C (I); 2E (I); 35C (II); 2E (II);
0.75			1C (I);		14C (I); 6E (I); 28C (II); 4E (II);
1					1E (I);
3		1E (I);			
6		1E (I);			
≥32		6C (I); 24C (II); 3E (II);			

**Table 3:** MIC data on ESBL *E. coli* resistance to ceftriaxone (CRO), ampicillin/sulbactam (AMS), amoxicillin/clavulanic acid (AUG), ciprofloxacin (CIP), gentamicin (GN), amikacin (AK), tetracycline (TE), aztreonam (ATM). C—number of ESBL *E. coli* isolated from cloacal swab samples. E—number of ESBL *E. coli* isolated from environmental swab samples. (I)—ESBL *E. coli* isolated from broiler production cycle I. (II)—ESBL *E. coli* isolated from broiler production cycle II. No color background—sensitive. Gray color background—resistant.

Antibiotic (μg/mL)	CRO	AMS	AUG	CIP	CN	AK	TE	ATM
0.094							1C (I);	
0.125					1C (I);			
0.19				2C (II);	1C (I);		2C (I);	
0.25					2C (I); 1C (II);		1C (I); 1C (II);	
0.38				1C (II);	3C (I); 8C (II); 2E (II);		2C (II);	
0.5					5C (I); 1E (I); 22C (II); 3E (II);		1C (II);	1C (I); 8C (II);
1			2C (I); 1E (II);		1C (I); 11C (II);	1C (I);	5C (I); 1E (I); 1C (II);	4C (I); 16C (II); 3E (II);
1.5		1C (I);	3C (I); 1E (I); 9C (II);	2C (I);	1E (I); 3C (II);	1C (I); 1C (II);	2C (I); 1E (I); 4C (II);	9C (I); 4E (I); 22C (II); 1E (II);
2	7C (II);	1C(II);	2C (I); 1E (I); 12C (II); 2E (II);	4C (I); 2E (I); 2C (II);	1C (II);	4C (I); 2E (I); 12C (II); 2E (II);		4C (I); 2E (I); 4C (II); 1E (II);
3	20C (II); 3E (II);	3C (II);	7C (I); 1E (I); 6C (II);	9C (I); 5E (I); 9C (II);		5C (I); 2E (I); 24C (II); 2E (II);		1C (II);
4	1C (I); 5C (II);	2C (I); 1E (I); 5C (II);	5C (I); 1E (I);	5C (I); 2E (I); 13C (II); 1E (II);		7C (I); 2E (I); 13C (II); 2E (II);		4C (II);
6	1E (I); 2C (II);	3C (I); 1E (I); 8C (II); 2E (II);	1C (II);	1C (I); 2C (II); 1E (II);		2C (I); 1E (I); 9C (II);		
8	5C (I); 1E (I);	4C (I); 1E (I); 10C (II);	1C (I);	1C (II);		1C (I); 4C (II);		1C (II);
24	5C (II);	1E (I); 6C (II);	1C (II);	1E (II);			1C (I); 2E (I); 8C (II); 2E (II);	1C (I);
≥32	1C (I); 4C (II);	8C (II);	3C (II); 1E (II);	1C (I); 27C (II); 3E (II);			1E (I); 7C (II);	1C (I);
48		1C (I); 2C (II); 1E (II);	4C (II); 1E (II);		1E (I);	1E (I);	9C (II);	1C (II);
64	1E (II);	1C (I); 2C (II);	8C (II);		2C (I);		2C (I); 10C (II); 1E (II);	1E (I);
96	2C (I); 1E (I);	1C (II);	7C (II);		1E (I);		1C (I); 4C (II);	
128	1C (I);	1C (II);	1C (II);		1C (I) 1E (I);		1C(I); 3C (II);	
192	1C (I); 1E (I); 1C (II);		1C (I); 2C (II);				2C (II);	
≥256	4C (I); 6C (II); 1E (II);	5C (I); 1E (I); 6C (II); 1E (II);	2C (I); 7C (II); 2E (II);			1C (I); 1C (II);	8C (II); 2E (II);	2C (I); 1E (I);

**Table 4:** Confirmed ESBL *E. coli* antimicrobial resistance profiles. CRO—Ceftriaxone. AMS—Ampicillin/sulbactam. AUG—Amoxicillin/clavulanic acid. CIP—Ciprofloxacin. MRP—Meropenem. CN—Gentamicin. AK—Amikacin. SXT—Trimethoprim/sulfamethoxazole. TE—Tetracycline. ATM—Aztreonam. IMI—Imipenem. FOS—Fosfomycin. CS—Colistin. C—Bacteria isolated from cloacal swab samples. E—Bacteria isolated from environmental swab samples.

Antimicrobial Resistance Profile	No of Total *E. coli* Strains (No of *E. coli* Isolated from Cloaca/No of *E. coli* Isolated from Environment)
CRO, CIP, TE	16 (14C/2E)
CRO, CIP, ATM	1C
CRO, CIP, AK	1E
CRO, CIP	18 (16C/2E)
CRO, AUG, CIP, TE	2C
CRO, AUG, CIP, SXT, TE	1C
CRO, AMS, SXT, TE	2C
CRO, AMS, CIP, TE	3C
CRO, AMS, CIP, CN, TE, ATM	1E
CRO, AMS, CIP, CN, SXT, TE, ATM	4 (2C/2E)
CRO, AMS, CIP, CN, AK, SXT, TE, ATM	1C
CRO, AMS, CIP, AK, TE	2 (1C/1E)
CRO, AMS, CIP, AK	1C
CRO, AMS, CIP	10 (8C/2E)
CRO, AMS, AUG, SXT, TE	1C
CRO, AMS, AUG, CIP, TE, ATM	1C
CRO, AMS, AUG, CIP, TE	8 (7C/1E)
CRO, AMS, AUG, CIP, SXT, TE, ATM	1C
CRO, AMS, AUG, CIP, SXT, TE	18 (15C/3E)
CRO, AMS, AUG, CIP, SXT	3C
CIP, TE	1C
AUG, CIP, TE	2C
AMS, AUG, CIP, SXT, TE	4C

## Data Availability

The original contributions presented in this study are included in the article. Further inquiries can be directed to the corresponding author.
